# Draft genome sequence of *Staphylococcus* sp. EZ-P03 isolated from a mesophilic anaerobic digester

**DOI:** 10.1186/s13104-018-3784-9

**Published:** 2018-10-03

**Authors:** Elvira E. Ziganshina, Waleed S. Mohammed, Elena I. Shagimardanova, Leyla H. Shigapova, Ayrat M. Ziganshin

**Affiliations:** 1 Department of Microbiology, Institute of Fundamental Medicine and Biology Kazan (Volga Region), Federal University, Kremlyovskaya STR. 18, Kazan, 420008 Russia; 2 Laboratory of Extreme Biology, Institute of Fundamental Medicine and Biology Kazan (Volga Region), Federal University, Kazan, 420021 Russia

**Keywords:** Draft genome, *Firmicutes*, *Staphylococcus* sp., Chicken manure, Laboratory-scale mesophilic reactor

## Abstract

**Objectives:**

*Staphylococcus* species of the family *Staphylococcaceae* are facultatively anaerobic Gram-positive cocci growing in clusters, pairs and occasionally in short chains. Staphylococci can be detected in different environments. They are common commensals, but some can also cause infections in humans. Hence, their investigation is required to understand ecology and genetics and to create an opportunity for comparative studies.

**Data description:**

In this study, we report the determination of a draft genome sequence of *Staphylococcus* sp. strain EZ-P03 which was isolated from anaerobically digested chicken waste materials. The draft genome of *Staphylococcus* sp. EZ-P03 constituted a total of 62 contigs (> 500 bp) amounting to 2,689,358 bp with a G+C content of 37.3% and a N50 contig size of 126,562 bp. The whole genome shotgun project of *Staphylococcus* sp. strain EZ-P03 has been deposited at DDBJ/ENA/GenBank under the accession number QPMO00000000.

## Objective

*Staphylococcus* species of the family *Staphylococcaceae* are facultatively anaerobic Gram-positive cocci growing in clusters, pairs and occasionally in short chains. Staphylococci grow by respiration or fermentation and can be found in different environmental niches. *S. aureus* and *S. epidermidis* species are common commensals but have a high pathogenic potential. *S. saprophyticus*, *S. haemolyticus*, *S. simulans*, *S. cohnii*, *S. warneri* and others can also cause infections in human [1, 2]. Some *Staphylococcus* species with proteolytic activities have been shown to survive in various anaerobic digesters [3, 4]. The anaerobic digestion process is an appropriate technology for different agricultural wastes utilization [5, 6]; however, it is also important to develop effective hygiene and sanitation procedures to minimize the potential disease transfer risk [7, 8]. Genome analysis of species belonging to the genus *Staphylococcus* is required to understand their ecology and genetics and to create an opportunity for comparative studies.

## Data description

*Staphylococcus* sp. EZ-P03 was isolated from a mesophilic anaerobic digester operated at high ammonia levels (> 6.0 NH_4_–N g L^−1^) and fed with chicken manure as monosubstrate [4]. The strain *Staphylococcus* sp. EZ-P03 was cultured at + 37 °C on Luria agar for 48 h under microaerophilic conditions. Genomic DNA from the bacterial cells (washed with sterile phosphate buffer solution) was extracted with the FastDNA spin kit (#116540600; MP Biomedicals). DNA quantification and quality control were performed by agarose gel electrophoresis and measurement with a NanoDrop 2000 spectrophotometer (#ND-2000; Thermo Fisher Scientific) confirming an optical density 260/280 of between 1.8 and 2 and finally stored at – 20 °C. The bacterial strain EZ-P03 was initially identified based on morphological, biochemical and growth characteristics and finally confirmed by sequencing of the 16S rRNA gene (Table 1) (16S rRNA gene sequence, 1404 bp, BLAST identity of 99% to *S. simulans* and 98% to *S. piscifermentans*, *S. condiment* and *S. carnosus*).

**Table 1:** Overview of data files/data sets

Label	Name of data file/data set	File types (file extension)	Data repository and identifier (DOI or accession number)
Data file 1	Whole genome shotgun project	FASTA	GenBank accession (QPMO00000000)
Data file 2	16S rRNA gene sequence	FASTA	GenBank accession (MH651712)

The paired-end DNA libraries were prepared as described previously by us [9] as well as in accordance with the Illumina protocol. DNA fragmentation and DNA library preparation were checked with a High Sensitivity DNA kit (#5067-4626; Agilent) and a 2100 Bioanalyzer (#G2939BA; Agilent). Genome sequencing was fulfilled with an Illumina MiSeq platform (#SY-410-1003; Illumina) at Joint KFU-Riken Laboratory, Kazan (Volga Region) Federal University (Kazan, Russia) as detailed previously [9]. A quality of the sequence data was analyzed with the FastQC software [10], the genome was assembled with the algorithm package Velvet, version 1.2.10 [11], and the arrangement of contigs was then achieved with the Mauve program, version 2.4.0 [12]. Rapid Annotation System Technology (RAST) server (annotation scheme: RASTtk) was used to annotate the whole genome sequence of *Staphylococcus* sp. strain EZ-P03 [13]. The rRNA and tRNA genes were predicted with the RNAmmer 1.2 [14] and tRNA scan-SE 1.23 [15], accordingly. PlasmidFinder-1.3 Server was used to identify plasmids in the sequenced strain [16]. Comparison of the genomic feature of *Staphylococcus* sp. strain EZ-P03 with some other *Staphylococcus* species was fulfilled with the data received from an integrated database EzBioCloud [17]. Finally, the strain was identified as *Staphylococcus* sp. belonging to the family *Staphylococcaceae* within the phylum *Firmicutes*.

The draft genome of *Staphylococcus* sp. strain EZ-P03 constituted a total of 62 contigs (> 500 bp) amounting to 2,689,358 bp with a G+C content of 37.3% and a N50 contig size of 126,562 bp. Two plasmid sequences were also identified in the strain EZ-P03 (3018 bp and 1111 bp). RAST server predicted 2571 coding sequences. The genome of *Staphylococcus* sp. strain EZ-P03 was shown to encode at least 68 RNAs, including 8 rRNAs and 60 tRNAs. The strain *Staphylococcus* sp. EZ-P03 has several genes responsible for saccharides and proteins degradation, mixed acid and lactate fermentation, as well as urea decomposition. Moreover, several genes responsible for resistance to antibiotics (such as fluoroquinolones) and toxic compounds, including mercury, arsenic, cadmium and chromium compounds, were observed.

## Limitations

The exact length of the genome, synteny, number of rRNA genes and repetitive elements cannot be reported since the obtained data is based on the draft level genome sequence.
